# The Allelopathic Activity of Aqueous Extracts of *Helianthus annuus* L., Grown in Boreal Conditions, on Germination, Development, and Physiological Indices of *Pisum sativum* L.

**DOI:** 10.3390/plants12091920

**Published:** 2023-05-08

**Authors:** Daiva Janusauskaite

**Affiliations:** Lithuanian Research Centre for Agriculture and Forestry, Institute of Agriculture, Department of Plant Nutrition and Agroecology, Instituto al. 1, Kėdainiai District, LT-58344 Akademija, Lithuania; daiva.janusauskaite@lammc.lt

**Keywords:** allelopathy, *Helianthus annuus*, vigor index, root/shoot ratio, inhibitory rate (IR), physiological traits, synthetic effect (SE), *Pisum sativum*

## Abstract

Evaluation of the allelopathy relationship between different crops is a sensible strategy fallowing the correct use of positive effects and to avoid the disadvantageous effects among plants. This study aimed to detect the allelopathic effect of aqueous extracts of sunflower (*Helianthus annuus* L.), grown in a boreal climatic zone, on the growth, subsequent development, and physiological traits of pea (*Pisum sativum* L.). Three factors have been studied: donor plant fertilization (unfertilized and fertilized), aqueous extracts from donor plant organs (leaves and stems (L+S), heads (H) and roots (R)); four concentrations of extracts (0%, 25%, 50% and 75%). The aqueous extracts from fertilized sunflower donor plant at 25% and 50% concentration acted as potential biostimulants that stimulated pea seed germination (SG), whereas L+S and R extracts at 75% concentration from unfertilized donor plant inhibited SG, at 4 days after sowing. The aqueous extracts demonstrated a stimulating effect on above-ground and root dry mass, compared to the control. The concentration of extracts demonstrated a significant inhibitory effect on SPAD. R extract revealed the strongest allelopathic effect on physiological traits of pea. L+S and H extracts at 25% concentration had stimulating effects, while 50% and 75% concentrations showed inhibiting effects on the photosynthetic rate. The water use efficiency, stomatal conductance, and stomatal limitation were inhibited, whereas, the transpiration rate, photosynthetic water use efficiency and intercellular CO_2_ concentration were stimulated, with an increasing of extract concentrations, comparatively to the control.

## 1. Introduction

Any direct or indirect inhibiting effect of one plant species on neighboring plants of another species through the release of phytotoxic compounds is denominated as allelopathy [1]. Allelopathy is one promising strategy that can be used in agroecosystems in a variety of ways, i.e., making good use of positive effects and avoiding negative effects on plants [2,3,4]. The allelochemicals can be either actively released by plant exudation [5] or passively produced during the decomposition of plant residues [6,7]. Most of allelochemicals are secondary plant metabolites, belonging to terpenoids, phenolic compounds, long chain fatty acids, organic cyanides, alkaloids, and others [8,9]. Allelopathic substances obtained by above-ground plant parts decomposing in soil could potentially influence a seedling’s growth [10,11,12,13], accumulation of dry mass [14,15] and photosynthesis [16,17,18].

Sunflower is known to intensely impact on the growth of surrounding plants because of its high allelopathic potential. More than 200 natural allelopathic compounds have been isolated so far from different cultivars of sunflower [19]. Most of the known allelochemicals of sunflower affect seed germination, root, and shoot development [18,20,21,22]. Sunflowers, grown within a crop rotation, have been found to exert allelopathic effects on weeds [23,24].

Allelochemicals are accumulated in all plant organs—roots, stems, leaves, flowers, fruits, and seeds [25,26]. Allelochemicals can also be extracted from allelopathic plants and used in sustainable agriculture [1,4,26].

Some crops grown after sunflowers are known to have lower than normal yields [7,27,28]. One reason for this may be allelochemical inhibition. Allelochemicals diminish nutrients and water absorption by roots [29] and suppress photosynthesis and respiration [3,30,31].

Laboratory experiments are very significant because they contribute to the evaluation of the allelopathic properties of the plant [32]. The most common laboratory bioassays for phytotoxic compounds are seed germination and growth experiments. If a sensitive test plant is exposed to allelopathic compounds, its seed germination and seedling growth are reduced [12,25].

Aqueous extracts of various organs of sunflower significantly suppress the growth of weeds [21,33,34] and agricultural plants [11,15,20,35]. It has been established that the amount of allelopathic compounds in a plant can be influenced by growing conditions and nutritional level [27]. The allelopathic potential of sunflower grown in the southern regions of Europe and the world is quite widely studied. However, as the climate changes and the growing areas of sunflower are moved to more northern regions, there are still unknowns, such as the amount of allelopathic compounds accumulated in sunflowers grown in the boreal climate zone. Additionally, the behavior of sunflowers in relation to other crop-rotation plants has not been studied in this zone. This is very important in order to include sunflowers in crop rotation for the purpose of crop-rotation diversification.

The present study was planned with the objectives (1) to evaluate the allelopathic potential of aqueous extracts of different organs of sunflower (*Helianthus annuus* L.) grown in the boreal climate zone on pea (*Pisum sativum* L.) germination and development, and (2) to find out the seedling growth, development, and alteration of physiological parameters of pea in response to sunflower allelopathy under field conditions.

## 2. Results

### 2.1. Effects of the Extracts on Pea Seed Germination (SG) 4 DAS

**Figure 1:**
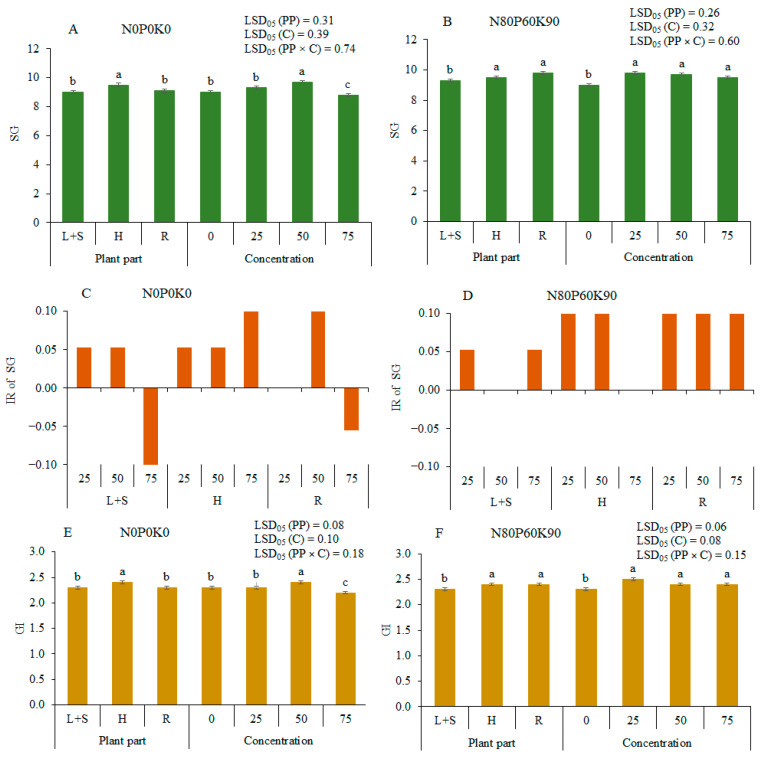
Allelopathic effects of different concentrations of the extracts of sunflower parts on seed germination (SG) ((**A**)—donor plant unfertilized, (**B**)—donor plant fertilized), inhibitory rate (IR) for SG (**C**,**D**) and germination index (GI) ((**E**)—donor plant unfertilized, (**F**)—donor plant fertilized) of pea 4 DAS. Different letters denote a statistically significant difference (at *p* ≤ 0.05 according to LSD) among treatments. The error bars show SE. L+S—leaves and steams; H—heads; R—roots. PP—plant part factor; C—concentration factor; PP × C—interaction of both factors.

The aqueous extracts of both unfertilized (N0P0K0) sunflower donor plant (UDP) and fertilized (N80P60K90) donor plant (FDP) stimulated seed germination SG and germination index (GI) in most cases, and the stimulating effect was significant (*p* < 0.05) (Figure 1A,B,E,F). The data, averaged across extract concentration, showed that under UDP, the head (H) and root (R) extracts stimulated SG by 5.6% and 1.1%, and under FDP, stimulated SG by 2.2% and 5.4%, respectively, compared with the (leaves and steams) L+S extract. The data, averaged across plant parts, revealed that all concentrations promoted SG in most cases, with one exception, when 75% concentration from UDP inhibited SG by 2.2%, compared with 0% (control).

The inhibitory rate (IR) for SG was higher than zero under all concentrations for FDP (Figure 1D). Meanwhile, under UDP, the L+S and R extracts were at 75% concentration, and IR was negative, indicating an inhibitory effect on SG (Figure 1C).

### 2.2. Effects of the Extracts on Root/Shoot Length Ratio and Vigour Index (VI)

**Figure 2:**
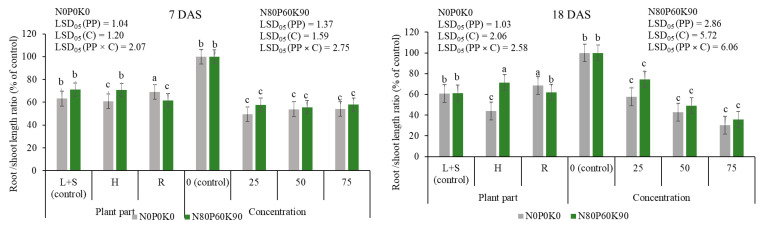
Effects of donor plant fertilization, plant part extracts and extracts concentration on root/shoot length ratio (% of control) of pea, 7 DAS and 18 DAS. L+S—leaves and steams; H—heads; R—roots. PP—plant part factor; C—concentration factor; PP × C—interaction of both factors. Different letters denote a statistically significant difference (at *p* ≤ 0.05 according to LSD) among treatments. The error bars show SE.

Figure 2 shows the present root/shoot length ratio in percent of the control. All extracts and concentrations of extracts showed a significant inhibitory effect on the root/shoot length ratio at 7 DAS and 18 DAS. The data, averaged across extract concentration at 7 DAS and 18 DAS, showed that H extract had a significant negative effect on the root/shoot length ratio, whereas R extract had a significant positive effect on this index, compared with L+S extract, under UDP. Under FDP, H extract revealed a significant positive effect at 18 DAS, compared with L+S extract. 

The data, averaged across donor plant parts at 18 DAS, revealed that the root/shoot length ratio decreased with increasing extract concentrations. 

**Figure 3:**
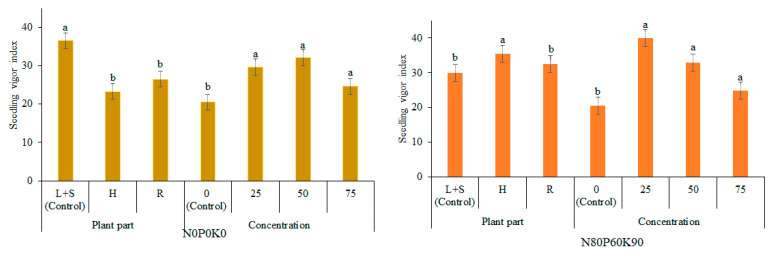
Effects of donor plant fertilization, plant part extracts and extract concentrations on vigor index of pea. The error bars show SE. L+S—leaves and steams; H—heads; R—roots. Different letters denote a statistically significant difference (at *p* ≤ 0.05 according to LSD) among treatments. The error bars show SE.

The application of extracts had a positive effect on the vigor index (VI) of pea (Figure 3). The data, averaged across extract concentration under UDP, showed that H and R extracts significantly inhibited VI by 36% and 27%, respectively, compared with L+S extract. The significant stimulating effect of H extract was found under FDP, when VI increased by 18%, in comparison with L+S extract. 

The data, averaged across donor plant parts, under both UDP and FDP extracts, showed that the VI was significantly higher by 25–40% and 21–96%, respectively, compared to the control (0%), but its values decreased with increasing concentrations.

### 2.3. Effects of the Extracts on Above-Ground Dry Mass (AGDM) and Root Dry Mass (RDM)

**Figure 4:**
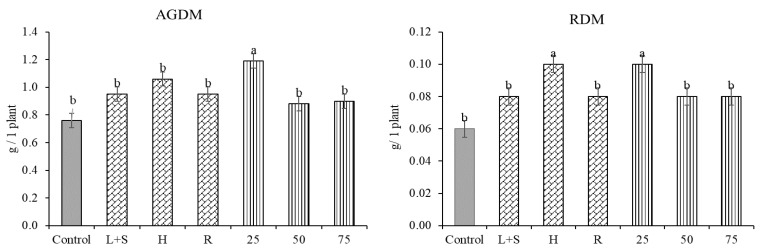
Effects of donor plant part and extracts concentrations on above-ground dry mass (AGDM) and root dry mass (RDM) of pea at flowering stage (averaged across donor plant fertilization). The error bars show SE. L+S—leaves and steams; H—heads; R—roots. Different letters denote a statistically significant difference (*p* ≤ 0.05 according to LSD) among treatments.

Field-planted plants from the rolls were dug up at the flowering stage, and the AGDM and RDM were evaluated. The data, averaged across donor plant fertilization, showed that the parts of donor plants and concentration of extracts stimulated both AGDM and RDM, compared to the control (Figure 4). H extract demonstrated the highest stimulating effect, when, compared with the control, AGDM and RDM increased by 40% and 59%, respectively. Meanwhile, the influence of L+S and R extracts was similar, and AGDM increased by 25% each, and RDM by 37% and 40%, respectively. UDP and FDP showed similar influences on AGDM and RDM.

Compared to the control, the lowest 25% concentration of extracts had the highest and significant stimulating effect and resulted in an increas in AGDM by 57% and RDM by 69%. The stimulating effect decreased with the increasing of the extract concentration, therefore 50% and 75% concentrations increased AGDM by 15–18% and RDM by 33–34%, in comparison with the control.

### 2.4. Effects of the Extracts on Physiological Parameters of Pea

Three-way ANOVA showed that the extract concentration (factor C), and interaction of fertilization of the donor plant and plant part (A × B) had a significant (*p* ≤ 0.05, *p* < 0.01) effect on the chlorophyll index (SPAD) in all measurements (Table 1). The influence of fertilization of the donor plant (factor A) on SPAD was significant (*p* ≤ 0.05) only at the BBCH 30 growth stage (GS), and the influence of interactions A × C and A × B × C was significant (*p* ≤ 0.01) at the BBCH 34–35 GS. The part of donor plant (factor B) did not have influence on the SPAD. The extracts concentration was the main factor that explained 22.6%, 57.9% and 18.5% of the total variability of SPAD in different GS. The influence of fertilization of the donor plant was significant (*p* ≤ 0.05) at the BBCH 30 GS and determined only 3.6% of the SPAD differences between treatments.

The data, averaged across fertilization of the donor plant and plant parts, showed that SPAD values progressively and significantly decreased as the concentration of extracts was increased. At 25%, 50% and 75% concentrations, SPAD was reduced by 5.6–24.8%, 14.4–31.4% and 12.9–29.3%, respectively, compared with the control.

The influence of donor plant fertilization (factor A) on Fv/Fm was significant (*p* ≤ 0.05, *p* < 0.01) in most cases, and it was found to be the main factor that explained 3.4–17.0% of the total Fv/Fm variability (Table 2). The concentration of the extract (factor C) significantly (*p* < 0.01) influenced Fv/Fm only at the BBCH 65 GS, but determined 6.3% of the differences of Fv/Fm only at the BBCH 65 GS. The effect of the plant part (factor B) on Fv/Fm was insignificant. The influence of the interaction of factors A × B, A × C and A × B × C on Fv/Fm was significant (*p* ≤ 0.05, *p* < 0.01) in some cases. 

Fv/Fm showed progressive decreases as the concentration of extract increased at BBCH 65 GS; Fv/Fm values diminished by 5.9%, 6.2% and 13.3%, respectively, at the 25%, 50% and 75% concentrations, compared to the control.

### 2.5. Effects of the Extracts on Gas Exchanges Parameters of Pea

Three-way ANOVA showed that all physiological traits were influenced by fertilization of the donor plant (factor A), donor plant part (factor B) and concentration of extract (factor C) (*p* ≤ 0.05, *p* < 0.01) in most cases (Table 3). The extract concentration was the main factor that explained 6.6–38.4% of the total variability of all physiological traits. The influence of plant parts was lower and governed 6.1–14.4% of physiological traits variation. Fertilization of the donor plant was responsible for only 4.6–10.9% of physiological parameter differences.

The data, averaged across donor plant part and extract concentration, revealed that fertilization of the donor plant had an inhibitory effect and decreased the photosynthetic rate (A), water use efficiency (WUE), photosynthetic water use efficiency (PWUE), mesophyll conductance (gm), and stomatal limitation value (Ls) by 33%, 34%, 53%, 42%, and 40%, respectively (Table 3). Meanwhile, the promoting effect of fertilization was established for transpiration rate (E), stomatal conductance (gs), and intercellular CO_2_ concentration (Ci), when values increased by 45%, 20% and 20%, respectively. 

The data, averaged across fertilization of the donor plant and extract concentration, demonstrated that the response of physiological traits of pea as affected by extracts from different parts of sunflower was varied. The lowest values of physiological traits were for plants treated with L+S extract. In comparison with L+S extract, R extract had the highest inhibitory effect on A, WUE, PWUE, gm and Ls, significantly reducing them by 38%, 41%, 65%, 67% and 53%, respectively. Meanwhile, R extract displayed a significant stimulating effect on E, gs and Ci and enhanced their values by 46%, 40% and 34%, respectively. The influence of H extract was less and was significant in only some cases. The ranking sequence of plant part treatments according to the allelopathic effect on physiological properties was L+S ˂ H ˂ R. 

The data, averaged across fertilization of the donor plant and donor plant part, revealed that the application of the extract at 25% concentration stimulated A by 24%, compared to the control (0%). When the concentration was increased to 50% and 75%, A was significantly inhibited by 19% and 28%, respectively (Table 3). 

With increasing of the concentration, WUE, gs and Ls were inhibited by 53–76%, 14–29% and 6–44%, respectively, whereas E, PWUE and gs were stimulated by 191–214%, 53–76% and 4–21%, compared to the control. 

The inhibitory rate (IR) evidenced the allelopathic effect of tested factors on physiological traits (Table 4). It was found that sunflower aqueous extracts at different concentrations had stimulating effects on E, gs and Ci, respectively, in 94%, 56% and 67% of the cases. Meanwhile, A, WUE, PWUE, gm, Ls, SPAD and Fv/Fm were inhibited in 78–94% of all tested cases.

**Figure 5:**
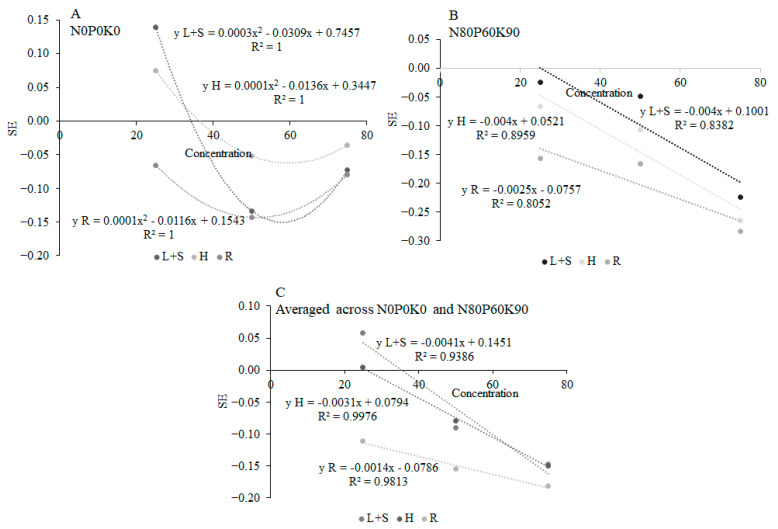
The non-linear fit of the synthetical effect (SE) for all physiological traits of pea under the influence of unfertilized sunflower aqueous extract (**A**), linear fit of SE under influence of fertilized sunflower aqueous extract (**B**), and linear fit of SE under influence of averaged across N0P0K0 and N80P60K90 (**C**), at concentrations 0%, 25%, 50% and 75%. L+S—leaves and steams; H—heads; R—roots.

Figure 5 shows the relationship between the synthetical effect (SE) for all physiological traits of pea and concentrations of sunflower aqueous extract. Under applying UDP extract, SE changed in a parabolic way through the application of 25%, 50% and 75% extract concentrations (Figure 5A). Under applying FDP extract, and under averaged UDP and FDP, SE changed in a linear way through the 25%, 50% and 75% extract concentrations (Figure 5B,C). Regarding SE values on physiological traits of pea, the trend L+S < H ˂ R was constant for both UDP and FDP, and in the case of their averages.

### 2.6. The Amount of Biologically Active Compounds 

**Figure 6:**
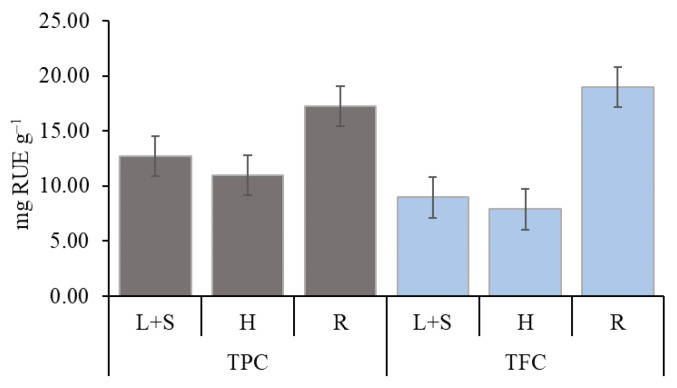
Total polyphenol (TPC) and total flavonoid (TFC) content in leaves and stems (L+S), heads (H) and roots (R) of sunflower extracts. The error bars show SE.

The total polyphenol content (TPC) in the extracts of sunflower organs varied from 10.96 to 17.24 mg RUE g^−1^ and total flavonoid content (TFC) ranged from 7.89 to 18.97 mg RUE g^−1^ (Figure 6). According to the amount of TPC and TFC, the extracts from different plant organs were arranged in the following ascending order: H < L+S < R. In comparison to H extract, which contained the least biologically active compounds, L+S extract contained by 16.2%, R extract by 57.3% more of TPC, and, respectively, by 13.7% and 140.4% more of TFC. 

## 3. Discussion

### 3.1. Effects of the Aqueous Extracts of Sunflower on Pea SG

Sunflower is an allelopathic plant that emits various biologically active chemical compounds, known as allelochemicals [8,19]. The differences in allelopathic potential between sunflower cultivars were found [13,23]. Varieties with high allelopathic activity through root exudates had the highest allelopathic effects through their residues [33]. The allelopathic substances released by plants are incorporated in soil and generated the harmful effects in the fields [36]. The sunflower residues contain secondary metabolites, which were release into the soil by the activity of microorganisms and affect the recipient plant [35]. The allelochemicals secreted by donor plants can inhibit seed germination, shoot growth and subsequent development in surrounding plants [7,23,27]. Therefore, it is important to know the effect of sunflower on the following crops in the next season. Allelopathic compounds released by leaching, root exudation, decomposition and volatilization have the same effect as the chemical herbicides of the weeds [37,38]. The seed germination rate evidences the amount of seed germination, and the germination index displays the germination capacity and vitality of the seeds [39]. These indicators are important on purpose to form an even crop of the required density. The results of the present investigation showed that the aqueous extracts of three sunflower organs (L+S, H, and R) had different effects on pea SG. In comparison with L+S extract, the highest stimulating effect on SG had H and R extracts from both UDP and FDP. Our experimental findings are in line with previous results [12,16,18], which indicate differences of the allelopathic effect among plant parts. These results are contrary to the study of Bashir et al. [11], wherein sunflower organ extracts inhibited wheat SG. However, the same researchers also reported that extracts of sunflower parts had different inhibitory effects: sunflower stem extract had the highest inhibitory effect on wheat SG, leaf extract was relatively less toxic, and root extract was the least toxic. Khaliq et al. [40] reported that the aqueous extract of sunflower significantly suppressed the seed germination of some weed species in rice. Various biologically active compounds have been found in sunflower organs [8,19], which are responsible for inhibiting or stimulating the SG and subsequent development of plants [10,11,16,17]. Using sunflower aqueous extracts, metabolic processes are markedly reduced or even stopped in germinating seeds, providing evidence for the rapid impact of certain allelochemicals on seed germination [41]. Sunflower stalks from which the extract is prepared can potentially be valorized as a source to produce biostimulants for improving salt stress tolerance in crops [42].

### 3.2. Effects of the Aqueous Extracts of Sunflower on Root/Shoot Length Ratio of Pea

The root/shoot length ratio is an important indicator for assessing plant health and is also a sensitive indicator of plant stress caused by chemical or physical factors. The results of the present experiment revealed that all aqueous extracts of three sunflower organs inhibited the root/shoot ratio as compared with the control. At 18 DAS, a general trend ascertained in all extracts of donor plant organs that the root/shoot length ratio decreased with increasing concentrations of the extracts. Similar results were reported by other researchers [15,43,44,45,46], where the aqueous extract of sunflower suppressed root/shoot length ratio of various crop plants. There are partially conflicting data that indicate that plant extracts significantly affected root and shoot growth, when the higher concentration showed the strongest inhibitory effect, whereas the lower concentration showed stimulatory effects in some cases [47].

### 3.3. Effects of the Aqueous Extracts of Sunflower on Pea AGDM and RDM

In comparison with control, all aqueous extracts demonstrated stimulating effect on AGDM and RDM of pea. H extract showed the highest promotion effect on these indices. Our experiment corroborates with previous results [12,16,20], in which it was found that allelopathic potential of sunflower parts vary from each other. Kandhro et al. [25] reported that shoot of sunflower was found more allelopathic than root.

The results of current experiment demonstrate that stimulating effect of sunflower aqueous extracts decreased with increasing of extract concentration. Our findings are in line with previous results [18,42], indicating that with the increase in concentration, the dry weights of rice, mung bean and chickpea were progressively reduced, compared to the control. Allelopathic compounds suppress water and nutrients uptake by roots, reducing photosynthesis and biomass accumulation [12,22]. The application of water extracts of sunflower reduced the weed dry weight (10–62.0%) compared to the control [48]. Sunflower extracts significantly reduced the dry mass of wheat and maize, respectively, by 31% and 34% compared to the control [15].

### 3.4. Effects of the Extracts of Sunflower on Physiological Traits of Pea

Photosynthesis is very sensitive to environmental changes, so photosynthetic indices are one of the most reliable indicators of plant stress. The applying of natural regulators, such as flavonoids and polyphenol compounds can act in growth as either stimulation or suppression, as well as affect biosynthesis, the photosynthetic pigments, and secondary metabolites in plants [49]. Chlorophyll is the essential pigment in the growth of plants, as they provide basic frameworks in photosynthesis. Stressful conditions negatively affect chlorophyll and can drastically reduce it in plant leaves [50]. The reduction of chlorophyll content in plants due to the application of allelopathic plant extracts was reported in many studies [20,21,51]. On the other hand, Kamal and Bano [16] investigated the effects of sunflower leaf, stem, and root extracts on chlorophyll accumulation in two varieties of wheat and found that sunflower leaf and root aqueous extracts significantly increased chlorophyll content in both varieties. It has been reported that allelopathic compounds have inhibitory effects on plant growth via influencing chlorophyll content and photosynthesis [3]. The results of current experiment showed that SPAD was significant decreased as the concentration of sunflower aqueous extracts was increased. The donor plant fertilization and sunflower organs did not affect SPAD. Our findings are consistent with the results of studies, conducted in habitual sunflower cultivation zones [17,43,45,46], wherein the extracts of sunflower were harmful to chlorophyll content and significantly reduced SPAD values in different plants.

The results of this experiment indicated that although all extracts from sunflower organs showed a certain degree of allelopathic activity, depending on both donor plant fertilization and extract concentration, several organs extract proved to possess greater overall inhibitory potential on physiological traits of pea. R extract revealed the strongest allelopathic effect on physiological indices. Our results agreed with those of the previous study [12] in that the extracts from the roots showed the greatest allelopathic effect and were ranged in the descending order of root, stem, and leaf. 

We found that the lowest concentration of extracts (25%) had a stimulating effect on A values. However, the influence of higher concentrations had a different allelopathic character. The reduction of photosynthesis, applying higher concentrations (50% and 75%) of aqueous extracts, could be influenced by the inhibition of root development, which made it difficult for water uptake and nutrient absorption of transplanted pea plants. The allelopathic compounds have suppressing effects that interfere with the physiological processes such as photosynthesis [3,52]. The same trend was noted in other studies [18,24].

The concentration of allelochemicals and allelopathic effects vary with the organ of the donor plant [1,15,35]. The results of the experiment demonstrate that the content of biologically active compounds in sunflower organs was unequal. The highest content of total polyphenols and total flavonoids was found in the R extract, and the lowest was in the H extract (H < L+S < R). This may be the reason why R extract demonstrated the strongest allelopathic effect on physiological traits of pea. Our results also supported the findings of Hussain et al. [53] and Tanase at al. [49] stated that there was remarkable reduction caused in plant physiological traits due to inhibitory phytotoxic compounds accumulated in donor plant parts. Changes in the photosynthetic activity of plants under the influence of allelopathic compounds also appeared in other studies [24,25]. Our results are in good agreement with Dadkhah [17], who documented that the photosynthesis (A) and stomatal conductance (gs) of plant recipients decreased with increasing concentrations of sunflower extract.

Sunflower, grown in crop rotation, may disconcert the growth process of neighboring plants, as a result of which the accumulation of assimilative substances and physiological processes under field conditions will decrease. Therefore, it is necessary to continue detailed research on the behavior of sunflowers, grown in the cool climate zone, in crop rotation. Knowledge on allelopathic interactions between sunflower and rotation plants could be used in sustainable weed management.

## 4. Materials and Methods

### 4.1. Details of the Laboratory Experiment

To reveal the allelopathic effects of sunflower, a laboratory experiment and field experiment at the Institute of Agriculture, Lithuanian Research Centre for Agriculture and Forestry (Lithuania, Kėdainiai district, 55°23′49″ N and 23°51′40″ E) were conducted. The locality is situated in a boreal climatic zone, with an average annual air temperature of 6.4 °C and a long-term annual precipitation of 568 mm. Pea (*Pisum sativum* L.), cultivar Respect, was as receptor crop, and sunflower (*Helianthus annus* L.), cultivar Peredovick, was as donor crop. Three factors have been studied—unfertilized donor plant (UDP) sunflower (N_0_P_0_K_0_) and fertilized (FDP) by N_80_P_60_K_90_ (Factor A); aqueous extract from different organs of plants: leaves and stems (L+S), heads (H) and roots (R) (Factor B); concentration: 0% *w*/*v*, 25% *w*/*v*, 50% *w*/*v* and 75% *w*/*v* (Factor C).

Fully mature donor plant sunflowers were collected from experimental fields of the Institute of Agriculture at the beginning of October 2020. Sunflower plants were separated into different organs (roots, heads, stems and leaves), which were cut into small pieces (10 mm) and air dried. L+S, R, and H powder (100 g of each) was added to 1.0 L deionized water and soaked for 24 h at room temperature (25 ± 2 °C).

The extracts were prepared by adding 100 g air-dried L+S/ or R/ or H in 1.0 L deionized water and soaked for 24 h at room temperature (25 ± 2 °C). The resultant extract was filtered using Whatman filter paper. This solution is considered as stock (standard) 100% solution. Then, extracts from different sunflower organs were diluted with deionized water from the stock solution to make the concentration of 25%, 50% and 75% (on the basis of volume) and stored in a refrigerator at 4 °C. The deionized water was used as control.

Seeds of the test plant are washed with deionized water. Seeds were germinated in a filter paper roll soaked with sunflower aqueous extracts as needed (20 mL of solution of the required concentration for each), so that the plants did not feel a lack of moisture. Three replicates of 10 seeds were performed for each treatment. Rolls of planted seeds are placed in containers and kept in the light at room temperature [54]. 

### 4.2. Determination of Biologically Active Compounds

Total phenol content (TPC) and total flavonoid content (TFC) in different organs of sunflower were determined according to spectrophotometric methods [55]. Results are expressed as mg of rutin equivalent (RUE) per 1 g of prepared sample. 

### 4.3. Data of Germination and Biometric Parameters

Four days after sowing (DAS), seed germination (SG) was recorded by counting the number of germinated seeds in each roll. A seed having 1 mm radicle was considered as germinated. Germination index (GI) was calculated according to the formula [39]:

(1) $$\mathrm{Germination}\;\mathrm{index}\;(\mathrm{GI})=\sum \;\frac{\mathrm{Gt}}{\mathrm{Dt}}$$

where Gt is the number of seeds emerging on a given day, and Dt is the time after setting the seeds for germination.

Root length (RL) and shoot length (SL) were measured by using a ruler and the root/shoot ratio was determined at 7 DAS and 18 DAS.

Seedling vigor index (VI) was calculated according to the formula:

VI = GI × S(2)

where GI is germination index, and S is the seedling length of a single seedling (mm) [56].

### 4.4. Details of the Field Experiment

This study was expanded to the field to evaluate the physiological effects of sunflower extracts on subsequent growth and development of pea. Then, 18 DAS in the rolls, the pea seedlings are transplanted into the field according scheme: Factor A—unfertilized donor plant (UDP) sunflower (N_0_P_0_K_0_) and fertilized (FDP) by N_80_P_60_K_90_; Factor B—aqueous extract from different organs of plants: leaves and stems (L+S), heads (H) and roots (R); Factor C—concentration: 0% *w*/*v*, 25% *w*/*v*, 50% *w*/*v* and 75% *w*/*v*. The study was performed in three replications, 10 plants in each. The row spacing 12 cm, the distance between plants in a row was 5 cm. Immediately after transplanting, the plants were watered once with water extracts of the appropriate concentration. Plants were grown outdoors for 48 days. The soil of the experimental site was Endocalcari-Epihypogleyic Cambisol. The soil was moderately rich in available phosphorus (100–125 mg kg^−1^, A-L method) and available potassium (140–170 mg kg^−1^, A-L method). The acidity was close to neutral (pH 6.5–6.8) (potentiometrically). The content of humus varied from low to moderate (1.8–2.2%) (Tyurin method).

### 4.5. Photosynthetic Performance and Accumulation of Dry Matter of Peas Planted in the Field

The physiological and gas exchange parameters were measured at the same time, randomly selected leaves of 10 plants per each line, from 10 am until 14 pm (local time) on clear days three-times per growing season: at the beginning of stem elongation (BBCH 30), at the middle of stem elongation (BBCH 34–35) and at full flowering stage (BBCH 65).

Relative chlorophyll content (SPAD index) was measured with a SPAD 502 (Minolta Camera Co. Ltd., Osaka, Japan).

The maximum quantum efficiency of PSII photochemistry (Fv/Fm) was measured using a multi-function pulse modulated handheld chlorophyll fluorometer (model OS-30p; Manufacturer: Opti-Sciences, Inc., Hudson, NH, USA). Fv/Fm was directly read after 20 min dark adaptation on the chlorophyll fluorometer [57]. The actinic light intensity was 1000 µmol m^−2^ s^−1^, modulation intensity two arbitrary units.

Leaf gas exchange measurements were performed using a portable infrared gas analyzer (SRS-1000) (ADC BioScientific Ltd., UK). The measurements were made on three randomly selected plants per plot on the first fully expanded leaf from the top. Photosynthetic parameters such as photosynthetic rate (A) (µmol m^−2^ s^−1^), transpiration rate (E) (mmol m^−2^ s^−1^), stomatal conductance (gs) (mol m^−2^ s^−1^) and intercellular CO_2_ concentration (Ci) (µmol mol^−1^) were recorded. The instantaneous water use efficiency (WUE) (µmol CO_2_ mmol^−1^ H_2_O) was calculated as A/E [58]. Photosynthetic water use efficiency (PWUE) (µmol CO_2_ mmol^−1^ H_2_O) was calculated as A/gs, and mesophyll conductance (gm) (mmol CO_2_ m^−2^ s^−1^) was calculated as A/Ci [59]. Stomatal limitation (Ls) was computed as Ls = 1 − (Ci/C0), where Ci is the intercellular CO_2_ concentration and C0 is the original CO_2_ concentration [58].

In each line, at full flowering stage (BBCH 65), 48 days after growing in the field, the plants were dug up, the above-ground part and roots were separated, and the samples were oven-dried at 80 °C until constant weight for dry mass. Samples were weighed using an electronic balance with an accuracy of 0.001 g. Depending on the number of plants in the row, the above-ground dry mass (AGDM) and root dry mass (RDM) of one plant were calculated.

The inhibitory rate (IR) was used to indicate the allelopathic effects of aqueous leaf extracts on seed germination (SG), germination index (GI) seedling growth, and physiological characteristics:

IR = 1 − C/T, if T ≥ C(3)

IR = T/C − 1, if T ˂ C(4)

where T is the treatment value and C is the control value [60].

Positive values of IR indicate a stimulatory effect, and negative values indicate an inhibitory effect of the aqueous extract. The synthetical allelopathic effect index (SE) was calculated as the arithmetic mean value of IR [60].

### 4.6. Statistical Analysis

Data of SG and GI were analyzed according to a factorial two-way ANOVA model (three plant parts for extract preparation (Factor A) and four concentrations of extracts (Factor B). Data of SPAD, Fv/Fm and all physiological parameters were analyzed according to a factorial three-way ANOVA model (2 fertilization levels of donor plant (Factor A), 3 plant parts for extract preparation (Factor B) and 4 concentrations of sunflower aqueous extracts (Factor C)). Homogeneity and normality were verified using Bartlett’s test. The data were compared using Fisher’s least significant difference (LSD) test at the probability levels *p* ≤ 0.05 and *p* ≤ 0.01. The statistical analysis was performed using Stat Eng from the statistical data processing package Selekcija.

## 5. Conclusions

The aqueous extracts from fertilized sunflower donor plant (FDP) at 25% and 50% concentrations acted as potential biostimulants stimulated pea seed germination (SG), whereas L+S and R extracts at 75% concentration from unfertilized donor plant (UDP) inhibited SG, at 4 days after sowing.

The application of extracts had a positive effect on the vigor index (VI) of pea.

Compared with L+S extract, H and R extracts under UDP significantly inhibited VI by 36% and 27%, respectively. Under FDP, H extracts significantly stimulated VI by 18%, in comparison with L+S extract. The extract concentration increased VI by 25–40% and 21–96%, respectively, under UDP and FDP extracts, compared to the control (0%), but VI decreased with increasing concentrations. The aqueous extracts from all parts of the donor plant and all concentrations demonstrated stimulating effect on above-ground dry mass (AGDM) and root dry mass (RDM) of pea, compared to the control. The stimulating effect decreased with increasing of extract concentrations.

The highest and significant inhibitory effect on SPAD was revealed by concentration of extracts. With increasing of extracts concentration, SPAD values decreased by 5.6–31.4%, compared to control (0%).

R extract showed the strongest allelopathic effect on physiological traits of pea. The lowest concentration (25%) of the L+S and H extracts revealed stimulating effects on A, while higher concentrations (50% and 75%) showed inhibiting effects (IR varied from −0.03 to −0.65). Compared to the control, WUE, gs and Ls were also inhibited, whereas E, PWUE and Ci were stimulated, with increasing extract concentrations.

## Figures and Tables

**Table 1:** Effects of donor plant fertilization, plant part, sunflower aqueous extract concentration on SPAD of pea in field.

Donor Plant	Extract from	Concentration,	SPAD		
Fertilization	Plant Part	% *w*/*v*	BBCH 30	BBCH 34–35	BBCH 65
Data, averaged across plant part and extract concentration					
N_0_P_0_K_0_			32.0 a	33.5 a	30.6 a
N_80_P_60_K_90_			30.4 b	33.6 a	31.7 a
Data, averaged across donor plant fertilization and extract concentration					
	L+S		30.9 a	33.3 a	31.1 a
	H		31.7 a	33.6 a	30.9 a
	R		31.0 a	33.9 a	31.5 a
Data, averaged across donor plant fertilization and plant part					
		0	34.0 a	42.7 a	35.9 a
		25	32.1 b	32.1 b	30.4 b
		50	29.1 b	29.3 b	30.3 b
		75	29.6 b	30.2 b	26.1 b
Contribution (% of sum of squares) of donor plant fertilization, plant part, aqueous extract concentration and their interaction and significance					
Fertilization (Factor A)			3.6 *	0.01	0.4
Plant part extract (Factor B)			0.7	0.1	0.1
Concentration (Factor C)			22.6 **	57.9 **	18.5 **
A × B			11.1 **	2.6 *	12.4 **
A × C			3.8	7.4 **	2.5
B × C			6.3	2.8	5.3
A × B × C			6.5	5.6 **	5.0
Total			54.9	76.4	34.3

BBCH scale (Biologische Bundesanstalt Bundessortenamt und Chemische Industrie). BBCH 30—beginning of stem elongation; BBCH 34– 35 –middle of stem elongation; BBCH 65—full flowering stage. L+S—leaves and steams; H—heads; R—roots. Different letters in column denote a statistically significant difference (LSD, *p* ≤ 0.05) among treatments. ** and * indicate statistical significance at *p* ≤ 0.01 and *p* ≤ 0.05, respectively.

**Table 2:** Effects of donor plant fertilization, plant part, sunflower aqueous extract concentration on Fv/Fm of pea in field.

Donor Plant	Extract from	Concentration,	Fv/Fm		
Fertilization	Plant Part	% *w*/*v*	BBCH 30	BBCH 34–35	BBCH 65
Data, averaged across plant part and extract concentration					
N_0_P_0_K_0_			0.797 a	0.665 a	0.651 a
N_80_P_60_K_90_			0.828 b	0.654 a	0.677 b
Data, averaged across donor plant fertilization and extract concentration					
	L+S		0.807 a	0.665 a	0.665 a
	H		0.820 b	0.658 a	0.666 a
	R		0.810 a	0.665 a	0.661 a
Data, averaged across donor plant fertilization and plant part					
		0	0.810 a	0.673 a	0.693 a
		25	0.809 a	0.667 a	0.652 b
		50	0.812 a	0.651 a	0.650 b
		75	0.817 a	0.646 a	0.601 b
Contribution (% of sum of squares) of donor plant fertilization, plant part, aqueous extract concentration and their interaction and significance					
Fertilization (Factor A)			17.0 **	0.7	3.4 *
Plant part extract (Factor B)			2.2	0.4	0.1
Concentration (Factor C)			0.7	2.9	6.3 **
A × B			18.3 **	6.4 *	1.1
A × C			5.9 **	2.8	4.0
B × C			5.3	8.2	6.8
A × B × C			9.8 **	10.9 *	4.3
Total			59.1	32.3	26.0

BBCH scale (Biologische Bundesanstalt Bundessortenamt und Chemische Industrie). BBCH 30—beginning of stem elongation; BBCH 34–35—middle of stem elongation; BBCH 65—full flowering stage. L+S—leaves and steams; H—heads; R—roots. Different letters in column denote a statistically significant difference (LSD, *p* ≤ 0.05) among treatments. ** and * indicate statistical significance at *p* ≤ 0.01 and *p* ≤ 0.05, respectively.

**Table 3:** Effects of donor plant fertilization, plant part, sunflower aqueous extract concentration on physiological traits of pea in field (averaged three measurements).

Donor Plant Fertilization	Extract from Plant Part	Concentration, % *w*/*v*	A	E	WUE	PWUE	gs	Ci	gm	Ls
Data, averaged across plant part and extract concentration										
N_0_P_0_K_0_			2.57 a	0.69 b	5.49 a	72.4 b	0.05 b	273 b	0.012 a	0.35 a
N_80_P_60_K_90_			1.72 b	1.00 a	3.62 b	33.9 c	0.06 a	327 a	0.007 b	0.21 b
Data, averaged across donor plant fertilization and extract concentration										
	L+S		2.70 a	0.70 b	5.92 a	81.9 b	0.05 b	256 b	0.015 a	0.38 a
	H		2.07 a	0.82 b	4.27 a	48.9 c	0.05 b	302 a	0.008 b	0.28 a
	R		1.68 b	1.02 a	3.48 b	28.8 c	0.07 a	342 a	0.005 b	0.18 b
Data, averaged across donor plant fertilization and plant part										
		0	2.28 a	0.36 b	8.8 a	33.9 c	0.07 b	276 b	0.090 a	0.34 a
		25	2.82 a	1.05 a	4.12 b	84.6 a	0.06 b	305 b	0.012 a	0.27 a
		50	1.84 b	0.85 a	3.22 b	51.3 b	0.05 c	286 b	0.010 a	0.32 a
		75	1.64 b	1.13 a	2.09 b	43.0 b	0.05 c	333 a	0.006 a	0.19 b
Contribution (% of sum of squares) of donor plant fertilization, plant part, aqueous extract concentration and their interaction and significance										
Fertilization (Factor A)			7.2 *	6.0 **	5.2 **	10.9 **	4.6 * c	8.5 **	6.6 *	8.6 **
Plant part (Factor B)			7.0 *	4.6	6.1 *	14.2 **	4.0	14.4 **	14.2 **	13.5 **
Concentration (Factor C)			8.2 *	22.7 **	38.4 **	10.8 **	6.6 *	5.5	3.8	6.7 *
A × B			1.7	3.6	3.7	6.2 *	4.9 *	5.3	3.3	4.4
A × C			5.0	2.2	4.3	9.7 **	2.6	3.0	6.0	3.1
B × C			5.6	6.9	3.9	8.0	6.5	8.1	6.9	8.0
A × B × C			1.9	2.3	1.8	7.3	7.6	3.7	4.2	3.4
Total			36.7	48.3	63.3	67.2	36.7	48.5	45.0	47.8

A—photosynthetic rate; E—transpiration rate; WUE—water use efficiency; PWUE—photosynthetic water use efficiency; gs—stomatal conductance; Ci—intercellular CO_2_ concentration; gm—mesophyll conductance; Ls—stomatal limitation value; L+S—leaves and steams; H—heads; R—roots. Different letters in column denote a statistically significant difference (LSD, *p* ≤ 0.05) among treatments. ** and * indicate statistical significance at *p ≤* 0.01 and *p ≤ 0.05*, respectively.

**Table 4:** The inhibitory rate (IR) for physiological traits of pea.

Plant Part	Concentration	IR_A_	IR_E_	IR_WUE_	IR_PWUE_	IR_gs_	IR_Ci_	IR_gm_	IR_Ls_	IR_SPAD_	IR_Fv/Fm_
N_0_P_0_K_0_											
L+S	25	0.45	0.38	0.06	−0.67	0.81	−0.25	0.50	0.38	−0.19	−0.07
	50	−0.23	−0.12	−0.17	−0.61	0.25	−0.21	−0.28	0.34	−0.22	−0.08
	75	−0.01	0.29	−0.19	−0.66	0.61	−0.18	−0.11	−0.10	−0.29	−0.08
H	25	0.28	0.28	−0.11	−0.59	0.67	−0.13	0.28	0.26	−0.12	−0.06
	50	−0.03	0.44	−0.61	−0.35	0.29	−0.04	0.02	0.02	−0.22	−0.04
	75	−0.02	0.65	−0.70	−0.32	0.21	0.03	−0.02	−0.01	−0.14	−0.04
R	25	−0.09	0.77	−0.80	0.07	−0.14	0.22	−0.24	−0.42	−0.01	−0.01
	50	−0.32	0.55	−0.77	−0.36	0.08	0.19	−0.43	−0.29	−0.04	−0.04
	75	−0.12	0.78	−0.81	0.05	−0.15	0.22	−0.22	−0.42	−0.13	−0.01
N_80_P_60_K_90_											
L+S	25	0.13	0.78	−0.74	0.11	−0.05	0.24	−0.09	−0.52	−0.09	−0.02
	50	−0.08	0.58	−0.60	−0.29	0.21	−0.18	0.10	−0.06	−0.16	−0.01
	75	−0.52	0.61	−0.78	−0.48	−0.10	0.20	−0.64	−0.48	−0.11	0.04
H	25	−0.14	0.50	−0.71	−0.24	0.22	0.18	−0.24	−0.29	0.04	0.02
	50	−0.16	0.62	−0.77	−0.28	0.09	0.18	−0.26	−0.28	−0.18	−0.02
	75	−0.56	0.78	−0.95	0.01	−0.56	0.29	−0.69	−0.77	−0.15	−0.04
R	25	−0.31	0.76	−0.85	−0.03	−0.22	0.21	−0.47	−0.45	−0.16	−0.05
	50	−0.48	0.38	−0.29	−0.01	−0.30	0.21	−0.53	−0.46	−0.17	−0.01
	75	−0.65	0.73	−0.90	−0.05	−0.40	0.26	−0.73	−0.59	−0.22	−0.29

A—photosynthetic rate; E—transpiration rate; WUE—water use efficiency; PWUE—photosynthetic water use efficiency; gs—stomatal conductance; Ci—intercellular CO_2_ concentration; gm—mesophyll conductance; Ls: stomatal limitation value; SPAD—chlorophyll index; Fv/Fm—maximum quantum efficiency of PSII. L+S—leaves and steams; H—heads; R—roots. The IR showing the inhibiting effect is marked with a grey background.

## Data Availability

Not applicable.
